# A tetra­nuclear chlorido-bridged manganese(II) cluster with imidazole ligands

**DOI:** 10.1107/S1600536808029139

**Published:** 2008-09-20

**Authors:** Mukhtar A. Kurawa, Christopher J. Adams, A. Guy. Orpen

**Affiliations:** a School of Chemistry, University of Bristol, Bristol, BS8 1TS, UK

## Abstract

The crystal structure of di-μ_3_-chlorido-tetra-μ_2_-chlorido-dichloridoocta­(imidazole-κ*N*)tetra­manganese(II) ethanol 1.234 solvate, [Mn_4_Cl_8_(C_3_H_4_N_2_)_8_]·1.234C_2_H_5_O or [Mn_4_Cl_8_(Him)_8_]·1.234EtOH, where Him is imidazole (C_3_H_4_N_2_), is based upon two Mn_4_Cl_4_ cubes which share one face, and which each lack one manganese vertex, giving a Mn_4_Cl_6_ unit. This contains two different octa­hedral coordination environments for the Mn atoms. Mn1 is coordinated by four bridging chlorido ligands and two imidazole N atoms, whereas Mn2 is coordinated by three bridging and one terminal Cl and two imidazole N atoms. The remaining two Mn centres are generated by inversion symmetry. A partial occupancy solvent mol­ecule (ethanol) is present. The crystal structure displays several N—H⋯Cl and N—H⋯O hydrogen bonds.

## Related literature

Lee *et al.* (2000 ▶) reported the structure and magnetic properties of a similar chlorido-bridged tetra­nuclear cluster of cadmium(II) with 2(2-pyrid­yl)-4,4′,5,5′-tetra­methyl-4,5-dihydro-1*H*-imidazol-1-oxy-3-*N*-oxide (NIToPY). For the structure of [{CdCl_2_(Him)_2_}_*n*_], see: Flook *et al.* (1973 ▶).
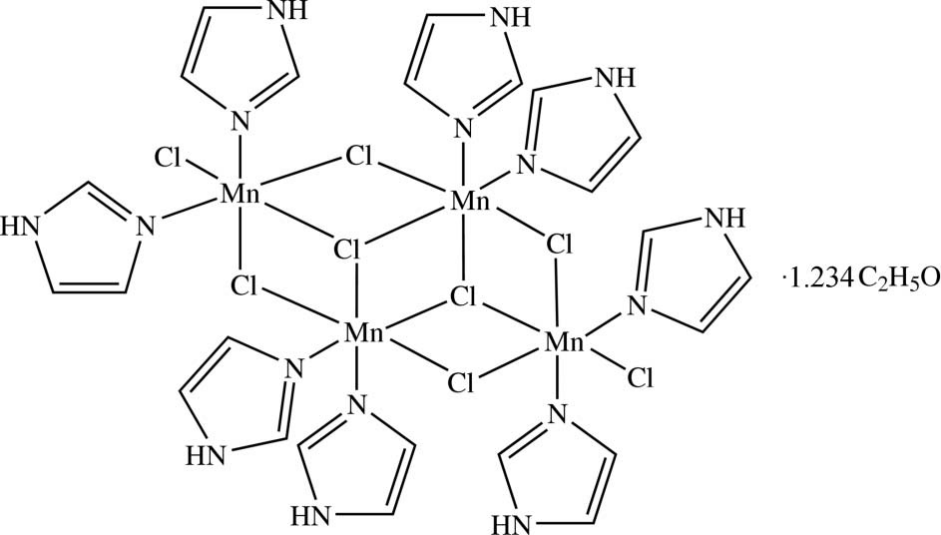


## Experimental

### 

#### Crystal data

[Mn_4_Cl_8_(C_3_H_4_N_2_)_8_]·1.234C_2_H_5_O
*M* *_r_* = 1103.45
Triclinic, 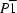

*a* = 8.4763 (16) Å
*b* = 10.696 (2) Å
*c* = 12.764 (2) Å
α = 99.206 (3)°
β = 105.706 (4)°
γ = 96.187 (3)°
*V* = 1085.7 (3) Å^3^
*Z* = 1
Mo *K*α radiation
μ = 1.68 mm^−1^
*T* = 173 (2) K
0.35 × 0.10 × 0.10 mm

#### Data collection

Bruker APEX CCD area-detector diffractometer
Absorption correction: multi-scan (*SADABS*; Sheldrick, 2008*a* ▶) *T*_min_ = 0.815, *T*_max_ = 0.850
11294 measured reflections
4920 independent reflections
3315 reflections with *I* > 2σ(*I*)
*R*_int_ = 0.048

#### Refinement

*R*[*F* ^2^ > 2σ(*F* ^2^)] = 0.058
*wR*(*F* ^2^) = 0.142
*S* = 1.04
4920 reflections
265 parameters
H-atom parameters constrained
Δρ_max_ = 1.17 e Å^−3^
Δρ_min_ = −0.77 e Å^−3^

### 

Data collection: *SMART* (Bruker, 2001 ▶); cell refinement: *SAINT* (Bruker, 2001 ▶); data reduction: *SAINT*; program(s) used to solve structure: *SHELXS97* (Sheldrick, 2008*b* ▶); program(s) used to refine structure: *SHELXL97* (Sheldrick, 2008*b* ▶); molecular graphics: *SHELXTL* (Sheldrick, 2008*b* ▶); software used to prepare material for publication: *SHELXTL*.

## Supplementary Material

Crystal structure: contains datablocks I, New_Global_Publ_Block. DOI: 10.1107/S1600536808029139/sg2263sup1.cif

Structure factors: contains datablocks I. DOI: 10.1107/S1600536808029139/sg2263Isup2.hkl

Additional supplementary materials:  crystallographic information; 3D view; checkCIF report

## Figures and Tables

**Table 1:** Hydrogen-bond geometry (Å, °)

*D*—H⋯*A*	*D*—H	H⋯*A*	*D*⋯*A*	*D*—H⋯*A*
N8—H8⋯O1^i^	0.88	2.09	2.865 (15)	147
N4—H6*A*⋯Cl3^ii^	0.88	2.49	3.289 (4)	152
N6—H10*A*⋯Cl4^ii^	0.88	2.48	3.247 (4)	146
N2—H2*A*⋯Cl4^iii^	0.88	2.55	3.292 (4)	143

Symmetry codes: (i) 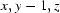; (ii) 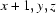; (iii) 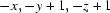.

**Fig. 1.**
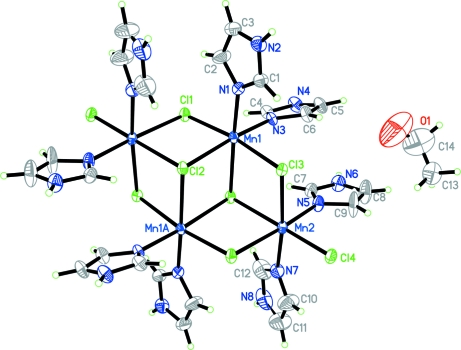
The molecular structure of I with atom labels and 50% probability displacement ellipsoids for non-H atoms. [Symmetry code (A):-x, -y, -z + 1]

**Fig. 2.**
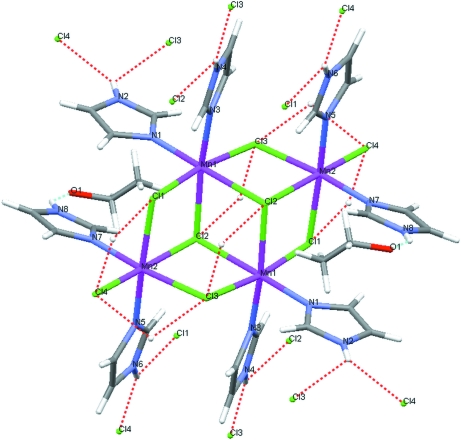
Hydrogen bond environment for I, showing only chlorine atoms of the neighbouring cluster.

## supplementary crystallographic information

### Comment

During our exploration of solid state routes for accessing coordination compounds, we ground CdCl_2_ with imidazole to form polymeric [{CdCl_2_(Him)_2_}_n_], whose structure was reported by Flook *et al.* (1973). We sought to synthesize the manganese analogue in a similar fashion by grinding MnCl_2_.4H_2_O with an equimolar amount of imidazole. The resulting polycrystalline powder (of unknown structure) was recrystallized from absolute ethanol to afford a single-crystal. The title compound I was obtained, which crystallizes in the triclinic crystal system in the *P*1 space group. The molecule is centrosymmetric, containing four manganese atoms in two different coordination environments and crystallizes across an inversion centre so there is a half a molecule in the asymmetric unit. One type of manganese atom is bonded to four bridging (two µ_2_-Cl and two µ_3_-Cl) chlorine atoms, and two imidazole N atoms, completing an octahedral coordination sphere. The second is type is bonded to three bridging (two µ_2_-Cl and one µ_3_-Cl) and one terminal chlorine atoms, again with two imidazole N atoms completing octahedral coordination. One of the imidazole ligands shows a carbon-nitrogen disorder, and a partial occupancy solvent molecule (ethanol) is present in the crystal structure, giving rise to N—H···O hydrogen bonds in addition to the N—H···Cl bonds between the chlorine atoms and the imidazole NH donors. The calculated powder pattern of I does not match that obtained from the crude material.

### Experimental

1 mmol of MnCl_2_.4H_2_O was ground with 2 mmol of imidazole resulting in the formation of an off-white polycrystalline powder. This was dissolved in absolute alcohol and the resulting solution allowed to evaporate slowly at room temperature, leading to the formation of colourless needle-like crystals.

### Refinement

H atoms were positioned geometrically and refined using a riding model, with C—H = 0.95 (2) Å and N—H = 0.88 (2) Å and *U*_iso_(H) = 1.2 times *U*_eq_(C, N) for the imidazole rings and C—H = 0.99 (2) Å and *U*_iso_(H) = 1.5 times *U*_eq_(C) for the ethanol molecule.

### Crystal data

**Table:**

[Mn_4_Cl_8_(C_3_H_4_N_2_)_8_]·1.234C_2_H_5_O	*Z* = 1
*M**_r_* = 1103.45	*F*(000) = 554.8
Triclinic, *P*1	*D*_x_ = 1.688 Mg m^−^^3^
*a* = 8.4763 (16) Å	Mo *K*α radiation, λ = 0.71073 Å
*b* = 10.696 (2) Å	Cell parameters from 3784 reflections
*c* = 12.764 (2) Å	θ = 2.3–27.5°
α = 99.206 (3)°	µ = 1.68 mm^−^^1^
β = 105.706 (4)°	*T* = 173 K
γ = 96.187 (3)°	Needle, colourless
*V* = 1085.7 (3) Å^3^	0.35 × 0.10 × 0.10 mm

### Data collection

**Table:**

Bruker APEX CCD area-detector diffractometer	4920 independent reflections
Radiation source: fine-focus sealed tube	3315 reflections with *I* > 2σ(*I*)
graphite	*R*_int_ = 0.048
phi and ω scans	θ_max_ = 27.5°, θ_min_ = 1.7°
Absorption correction: multi-scan (SADABS; Sheldrick, 2008a)	*h* = −11→10
*T*_min_ = 0.815, *T*_max_ = 0.850	*k* = −13→13
11294 measured reflections	*l* = −16→16

### Refinement

**Table:**

Refinement on *F*^2^	Primary atom site location: structure-invariant direct methods
Least-squares matrix: full	Secondary atom site location: difference Fourier map
*R*[*F*^2^ > 2σ(*F*^2^)] = 0.058	Hydrogen site location: inferred from neighbouring sites
*wR*(*F*^2^) = 0.142	H-atom parameters constrained
*S* = 1.04	*w* = 1/[σ^2^(*F*_o_^2^) + (0.055*P*)^2^ + 2.417*P*] where *P* = (*F*_o_^2^ + 2*F*_c_^2^)/3
4920 reflections	(Δ/σ)_max_ < 0.001
265 parameters	Δρ_max_ = 1.17 e Å^−^^3^
0 restraints	Δρ_min_ = −0.77 e Å^−^^3^

### Special details

**Table:**

Geometry. All e.s.d.'s (except the e.s.d. in the dihedral angle between two l.s. planes) are estimated using the full covariance matrix. The cell e.s.d.'s are taken into account individually in the estimation of e.s.d.'s in distances, angles and torsion angles; correlations between e.s.d.'s in cell parameters are only used when they are defined by crystal symmetry. An approximate (isotropic) treatment of cell e.s.d.'s is used for estimating e.s.d.'s involving l.s. planes.
Refinement. Refinement of *F*^2^ against ALL reflections. The weighted *R*-factor *wR* and goodness of fit *S* are based on *F*^2^, conventional *R*-factors *R* are based on *F*, with *F* set to zero for negative *F*^2^. The threshold expression of *F*^2^ > σ(*F*^2^) is used only for calculating *R*-factors(gt) *etc*. and is not relevant to the choice of reflections for refinement. *R*-factors based on *F*^2^ are statistically about twice as large as those based on *F*, and *R*- factors based on ALL data will be even larger.

### Fractional atomic coordinates and isotropic or equivalent isotropic displacement parameters (Å^2^)

**Table:**

	*x*	*y*	*z*	*U*_iso_*/*U*_eq_	Occ. (<1)
Mn1	0.11612 (8)	0.13627 (6)	0.44334 (5)	0.01743 (18)	
Mn2	0.14342 (8)	0.17089 (6)	0.75623 (5)	0.01930 (19)	
Cl1	0.15783 (14)	−0.04351 (11)	0.30310 (9)	0.0227 (3)	
Cl2	−0.18410 (13)	0.00684 (10)	0.40027 (9)	0.0191 (2)	
Cl3	0.05611 (13)	0.29485 (10)	0.59545 (9)	0.0194 (2)	
Cl4	0.07690 (15)	0.33793 (11)	0.88887 (9)	0.0258 (3)	
N1	0.0440 (5)	0.2621 (4)	0.3243 (3)	0.0242 (9)	
N2	−0.0167 (5)	0.4279 (4)	0.2492 (3)	0.0319 (10)	
H2A	−0.0456	0.5033	0.2418	0.038*	
N3	0.3775 (5)	0.2228 (3)	0.4924 (3)	0.0208 (8)	
N4	0.6483 (5)	0.2509 (4)	0.5285 (4)	0.0313 (10)	
H6A	0.7486	0.2343	0.5312	0.038*	
N5	0.4062 (5)	0.2587 (4)	0.7973 (3)	0.0273 (9)	
N6	0.6744 (5)	0.2843 (4)	0.8230 (4)	0.0339 (10)	
H10A	0.7720	0.2686	0.8176	0.041*	
N7	0.2169 (5)	0.0477 (4)	0.8769 (3)	0.0305 (10)	
C11	0.2455 (11)	−0.0398 (8)	1.0273 (6)	0.093 (4)	0.14 (9)
H11	0.2351	−0.0564	1.0963	0.111*	
N8'	0.2455 (11)	−0.0398 (8)	1.0273 (6)	0.093 (4)	0.86 (9)
C1	−0.0020 (6)	0.3753 (5)	0.3382 (4)	0.0276 (11)	
H1A	−0.0224	0.4151	0.4043	0.033*	
C2	0.0570 (9)	0.2431 (6)	0.2190 (4)	0.0452 (16)	
H4B	0.0877	0.1684	0.1835	0.054*	
C3	0.0209 (8)	0.3439 (5)	0.1723 (5)	0.0419 (15)	
H3B	0.0216	0.3543	0.0999	0.050*	
C4	0.5062 (6)	0.1690 (5)	0.4816 (4)	0.0274 (11)	
H5A	0.4992	0.0830	0.4451	0.033*	
C5	0.6102 (6)	0.3629 (5)	0.5705 (5)	0.0346 (13)	
H7B	0.6861	0.4390	0.6082	0.042*	
C6	0.4431 (6)	0.3469 (5)	0.5491 (4)	0.0280 (11)	
H8A	0.3811	0.4105	0.5697	0.034*	
C7	0.5294 (6)	0.2089 (5)	0.7709 (4)	0.0266 (11)	
H9A	0.5164	0.1296	0.7212	0.032*	
C8	0.6458 (8)	0.3888 (6)	0.8853 (6)	0.0563 (19)	
H11A	0.7259	0.4591	0.9312	0.068*	
C9	0.4800 (8)	0.3732 (6)	0.8693 (6)	0.060 (2)	
H12B	0.4230	0.4321	0.9026	0.072*	
C10	0.1834 (11)	0.0537 (8)	0.9716 (6)	0.071 (2)	
H13A	0.1235	0.1149	0.9989	0.085*	
N8	0.3293 (11)	−0.1058 (8)	0.9569 (8)	0.079 (4)	0.14 (9)
H8	0.3828	−0.1704	0.9700	0.095*	
C11'	0.3293 (11)	−0.1058 (8)	0.9569 (8)	0.079 (4)	0.86 (9)
C12	0.3115 (11)	−0.0513 (9)	0.8673 (8)	0.090 (3)	
H16A	0.3556	−0.0757	0.8075	0.108*	
O1	0.5305 (15)	0.7035 (11)	0.9177 (10)	0.111 (4)	0.617 (10)
C13	0.443 (2)	0.6632 (16)	0.7342 (11)	0.097 (6)	0.617 (10)
H17A	0.3723	0.7299	0.7260	0.145*	0.617 (10)
H18B	0.3765	0.5830	0.7359	0.145*	0.617 (10)
H19C	0.4887	0.6497	0.6713	0.145*	0.617 (10)
C14	0.548 (3)	0.694 (2)	0.8143 (16)	0.128 (8)	0.617 (10)
H20A	0.6028	0.7797	0.8117	0.154*	0.617 (10)
H21B	0.6291	0.6350	0.8098	0.154*	0.617 (10)

### Atomic displacement parameters (Å^2^)

**Table:**

	*U*^11^	*U*^22^	*U*^33^	*U*^12^	*U*^13^	*U*^23^
Mn1	0.0148 (4)	0.0163 (4)	0.0199 (4)	0.0015 (3)	0.0049 (3)	0.0011 (3)
Mn2	0.0151 (4)	0.0200 (4)	0.0188 (4)	0.0021 (3)	0.0003 (3)	0.0009 (3)
Cl1	0.0185 (6)	0.0204 (6)	0.0263 (6)	0.0020 (5)	0.0062 (5)	−0.0021 (4)
Cl2	0.0148 (6)	0.0177 (5)	0.0223 (5)	0.0032 (4)	0.0025 (4)	0.0014 (4)
Cl3	0.0173 (6)	0.0194 (6)	0.0200 (5)	0.0041 (4)	0.0040 (4)	0.0013 (4)
Cl4	0.0244 (6)	0.0255 (6)	0.0237 (6)	0.0007 (5)	0.0069 (5)	−0.0036 (5)
N1	0.026 (2)	0.025 (2)	0.0202 (19)	0.0022 (18)	0.0055 (17)	0.0034 (16)
N2	0.036 (3)	0.024 (2)	0.035 (2)	0.008 (2)	0.006 (2)	0.0108 (19)
N3	0.017 (2)	0.016 (2)	0.030 (2)	0.0015 (16)	0.0058 (17)	0.0050 (16)
N4	0.011 (2)	0.036 (3)	0.046 (3)	0.0024 (18)	0.0052 (19)	0.012 (2)
N5	0.016 (2)	0.028 (2)	0.030 (2)	0.0015 (18)	0.0011 (17)	−0.0047 (18)
N6	0.016 (2)	0.042 (3)	0.038 (2)	0.006 (2)	0.0026 (19)	0.003 (2)
N7	0.031 (3)	0.030 (2)	0.027 (2)	0.006 (2)	−0.0024 (19)	0.0121 (18)
C11	0.119 (7)	0.097 (7)	0.067 (5)	0.025 (6)	0.014 (5)	0.049 (5)
N8'	0.119 (7)	0.097 (7)	0.067 (5)	0.025 (6)	0.014 (5)	0.049 (5)
C1	0.037 (3)	0.021 (3)	0.028 (3)	0.012 (2)	0.013 (2)	0.007 (2)
C2	0.081 (5)	0.034 (3)	0.030 (3)	0.019 (3)	0.028 (3)	0.008 (2)
C3	0.065 (4)	0.035 (3)	0.029 (3)	0.004 (3)	0.019 (3)	0.010 (2)
C4	0.020 (3)	0.027 (3)	0.036 (3)	0.006 (2)	0.007 (2)	0.009 (2)
C5	0.021 (3)	0.031 (3)	0.048 (3)	−0.005 (2)	0.008 (2)	0.008 (2)
C6	0.020 (3)	0.022 (3)	0.040 (3)	0.004 (2)	0.008 (2)	0.004 (2)
C7	0.020 (3)	0.030 (3)	0.026 (2)	0.007 (2)	0.003 (2)	0.002 (2)
C8	0.026 (3)	0.052 (4)	0.069 (4)	−0.004 (3)	0.004 (3)	−0.025 (3)
C9	0.033 (4)	0.045 (4)	0.081 (5)	−0.009 (3)	0.017 (3)	−0.038 (3)
C10	0.103 (7)	0.069 (5)	0.053 (4)	0.032 (5)	0.025 (4)	0.031 (4)
N8	0.076 (6)	0.059 (5)	0.087 (7)	0.011 (4)	−0.011 (5)	0.033 (5)
C11'	0.076 (6)	0.059 (5)	0.087 (7)	0.011 (4)	−0.011 (5)	0.033 (5)
C12	0.075 (6)	0.087 (7)	0.091 (6)	−0.005 (5)	−0.014 (5)	0.050 (6)
O1	0.111 (10)	0.106 (9)	0.117 (9)	0.045 (7)	0.025 (8)	0.015 (7)
C13	0.145 (17)	0.109 (13)	0.043 (8)	0.071 (12)	0.023 (9)	0.009 (8)
C14	0.15 (2)	0.18 (2)	0.097 (14)	0.066 (17)	0.072 (15)	0.041 (15)

### Geometric parameters (Å, °)

**Table:**

Mn1—N3	2.186 (4)	N6—H10A	0.8800
Mn1—N1	2.196 (4)	N7—C10	1.308 (8)
Mn1—Cl1	2.5328 (13)	N7—C12	1.405 (10)
Mn1—Cl3	2.5636 (13)	C11—C10	1.387 (10)
Mn1—Cl2	2.6332 (13)	C11—N8	1.436 (11)
Mn1—Cl2^i^	2.6841 (13)	C11—H11	0.9500
Mn2—N7	2.192 (4)	C1—H1A	0.9500
Mn2—N5	2.208 (4)	C2—C3	1.338 (8)
Mn2—Cl4	2.4784 (14)	C2—H4B	0.9500
Mn2—Cl3	2.6033 (13)	C3—H3B	0.9500
Mn2—Cl1^i^	2.6121 (14)	C4—H5A	0.9500
Mn2—Cl2^i^	2.6498 (13)	C5—C6	1.355 (7)
Cl1—Mn2^i^	2.6121 (14)	C5—H7B	0.9500
Cl2—Mn2^i^	2.6498 (13)	C6—H8A	0.9500
Cl2—Mn1^i^	2.6841 (13)	C7—H9A	0.9500
N1—C1	1.311 (6)	C8—C9	1.353 (8)
N1—C2	1.364 (6)	C8—H11A	0.9500
N2—C1	1.329 (6)	C9—H12B	0.9500
N2—C3	1.349 (7)	C10—H13A	0.9500
N2—H2A	0.8800	N8—C12	1.345 (10)
N3—C4	1.316 (6)	N8—H8	0.8800
N3—C6	1.382 (6)	C12—H16A	0.9500
N4—C4	1.338 (6)	O1—C14	1.356 (18)
N4—C5	1.341 (7)	C13—C14	1.13 (2)
N4—H6A	0.8800	C13—H17A	0.9800
N5—C7	1.319 (6)	C13—H18B	0.9800
N5—C9	1.378 (7)	C13—H19C	0.9800
N6—C7	1.332 (6)	C14—H20A	0.9900
N6—C8	1.349 (7)	C14—H21B	0.9900
			
N3—Mn1—N1	92.56 (14)	C7—N6—H10A	125.9
N3—Mn1—Cl1	92.29 (10)	C8—N6—H10A	125.9
N1—Mn1—Cl1	95.12 (11)	C10—N7—C12	107.3 (6)
N3—Mn1—Cl3	92.37 (10)	C10—N7—Mn2	126.6 (5)
N1—Mn1—Cl3	90.64 (11)	C12—N7—Mn2	126.1 (5)
Cl1—Mn1—Cl3	172.42 (5)	C10—C11—N8	104.1 (7)
N3—Mn1—Cl2	171.84 (10)	C10—C11—H11	128.0
N1—Mn1—Cl2	95.45 (11)	N8—C11—H11	128.0
Cl1—Mn1—Cl2	85.48 (4)	N1—C1—N2	112.2 (4)
Cl3—Mn1—Cl2	89.08 (4)	N1—C1—H1A	123.9
N3—Mn1—Cl2^i^	90.21 (10)	N2—C1—H1A	123.9
N1—Mn1—Cl2^i^	174.19 (11)	C3—C2—N1	110.8 (5)
Cl1—Mn1—Cl2^i^	89.87 (4)	C3—C2—H4B	124.6
Cl3—Mn1—Cl2^i^	84.14 (4)	N1—C2—H4B	124.6
Cl2—Mn1—Cl2^i^	81.95 (4)	C2—C3—N2	105.9 (5)
N7—Mn2—N5	89.13 (16)	C2—C3—H3B	127.0
N7—Mn2—Cl4	94.57 (12)	N2—C3—H3B	127.0
N5—Mn2—Cl4	94.27 (11)	N3—C4—N4	111.4 (4)
N7—Mn2—Cl3	173.40 (12)	N3—C4—H5A	124.3
N5—Mn2—Cl3	91.82 (11)	N4—C4—H5A	124.3
Cl4—Mn2—Cl3	91.88 (4)	N4—C5—C6	107.0 (5)
N7—Mn2—Cl1^i^	88.65 (12)	N4—C5—H7B	126.5
N5—Mn2—Cl1^i^	173.48 (11)	C6—C5—H7B	126.5
Cl4—Mn2—Cl1^i^	92.01 (4)	C5—C6—N3	108.8 (5)
Cl3—Mn2—Cl1^i^	89.69 (4)	C5—C6—H8A	125.6
N7—Mn2—Cl2^i^	89.40 (12)	N3—C6—H8A	125.6
N5—Mn2—Cl2^i^	90.27 (11)	N5—C7—N6	111.2 (4)
Cl4—Mn2—Cl2^i^	174.01 (5)	N5—C7—H9A	124.4
Cl3—Mn2—Cl2^i^	84.06 (4)	N6—C7—H9A	124.4
Cl1^i^—Mn2—Cl2^i^	83.58 (4)	N6—C8—C9	106.0 (5)
Mn1—Cl1—Mn2^i^	97.01 (4)	N6—C8—H11A	127.0
Mn1—Cl2—Mn2^i^	93.69 (4)	C9—C8—H11A	127.0
Mn1—Cl2—Mn1^i^	98.05 (4)	C8—C9—N5	109.6 (5)
Mn2^i^—Cl2—Mn1^i^	93.71 (4)	C8—C9—H12B	125.2
Mn1—Cl3—Mn2	97.74 (4)	N5—C9—H12B	125.2
C1—N1—C2	104.0 (4)	N7—C10—C11	112.0 (8)
C1—N1—Mn1	130.2 (3)	N7—C10—H13A	124.0
C2—N1—Mn1	125.4 (4)	C11—C10—H13A	124.0
C1—N2—C3	107.1 (4)	C12—N8—C11	107.9 (8)
C1—N2—H2A	126.4	C12—N8—H8	126.1
C3—N2—H2A	126.4	C11—N8—H8	126.1
C4—N3—C6	105.2 (4)	N8—C12—N7	108.7 (9)
C4—N3—Mn1	128.8 (3)	N8—C12—H16A	125.7
C6—N3—Mn1	125.9 (3)	N7—C12—H16A	125.7
C4—N4—C5	107.7 (4)	C13—C14—O1	125 (2)
C4—N4—H6A	126.2	C13—C14—H20A	106.1
C5—N4—H6A	126.2	O1—C14—H20A	106.1
C7—N5—C9	105.0 (4)	C13—C14—H21B	106.1
C7—N5—Mn2	128.7 (3)	O1—C14—H21B	106.1
C9—N5—Mn2	125.9 (4)	H20A—C14—H21B	106.3
C7—N6—C8	108.2 (5)		
			
N3—Mn1—Cl1—Mn2^i^	175.98 (10)	N7—Mn2—N5—C9	−98.3 (5)
N1—Mn1—Cl1—Mn2^i^	−91.24 (11)	Cl4—Mn2—N5—C9	−3.8 (5)
Cl2—Mn1—Cl1—Mn2^i^	3.85 (4)	Cl3—Mn2—N5—C9	88.3 (5)
Cl2^i^—Mn1—Cl1—Mn2^i^	85.78 (4)	Cl2^i^—Mn2—N5—C9	172.3 (5)
N1—Mn1—Cl2—Mn2^i^	90.95 (11)	N5—Mn2—N7—C10	110.9 (6)
Cl1—Mn1—Cl2—Mn2^i^	−3.77 (4)	Cl4—Mn2—N7—C10	16.7 (6)
Cl3—Mn1—Cl2—Mn2^i^	−178.49 (4)	Cl1^i^—Mn2—N7—C10	−75.2 (6)
Cl2^i^—Mn1—Cl2—Mn2^i^	−94.28 (4)	Cl2^i^—Mn2—N7—C10	−158.8 (6)
N1—Mn1—Cl2—Mn1^i^	−174.77 (11)	N5—Mn2—N7—C12	−68.0 (5)
Cl1—Mn1—Cl2—Mn1^i^	90.51 (4)	Cl4—Mn2—N7—C12	−162.2 (5)
Cl3—Mn1—Cl2—Mn1^i^	−84.21 (4)	Cl1^i^—Mn2—N7—C12	105.9 (5)
Cl2^i^—Mn1—Cl2—Mn1^i^	0.0	Cl2^i^—Mn2—N7—C12	22.3 (5)
N3—Mn1—Cl3—Mn2	−85.49 (10)	C2—N1—C1—N2	−0.6 (6)
N1—Mn1—Cl3—Mn2	−178.08 (11)	Mn1—N1—C1—N2	172.4 (3)
Cl2—Mn1—Cl3—Mn2	86.47 (4)	C3—N2—C1—N1	0.4 (6)
Cl2^i^—Mn1—Cl3—Mn2	4.47 (4)	C1—N1—C2—C3	0.6 (7)
N5—Mn2—Cl3—Mn1	85.55 (11)	Mn1—N1—C2—C3	−172.9 (4)
Cl4—Mn2—Cl3—Mn1	179.89 (5)	N1—C2—C3—N2	−0.4 (8)
Cl1^i^—Mn2—Cl3—Mn1	−88.11 (4)	C1—N2—C3—C2	0.0 (7)
Cl2^i^—Mn2—Cl3—Mn1	−4.53 (4)	C6—N3—C4—N4	0.4 (5)
N3—Mn1—N1—C1	−89.6 (5)	Mn1—N3—C4—N4	−175.7 (3)
Cl1—Mn1—N1—C1	177.8 (4)	C5—N4—C4—N3	−0.6 (6)
Cl3—Mn1—N1—C1	2.8 (4)	C4—N4—C5—C6	0.5 (6)
Cl2—Mn1—N1—C1	91.9 (4)	N4—C5—C6—N3	−0.2 (6)
N3—Mn1—N1—C2	82.0 (5)	C4—N3—C6—C5	−0.1 (5)
Cl1—Mn1—N1—C2	−10.5 (5)	Mn1—N3—C6—C5	176.2 (3)
Cl3—Mn1—N1—C2	174.4 (5)	C9—N5—C7—N6	0.8 (6)
Cl2—Mn1—N1—C2	−96.5 (5)	Mn2—N5—C7—N6	−171.6 (3)
N1—Mn1—N3—C4	−117.3 (4)	C8—N6—C7—N5	−0.7 (6)
Cl1—Mn1—N3—C4	−22.1 (4)	C7—N6—C8—C9	0.3 (8)
Cl3—Mn1—N3—C4	151.9 (4)	N6—C8—C9—N5	0.2 (9)
Cl2^i^—Mn1—N3—C4	67.8 (4)	C7—N5—C9—C8	−0.6 (8)
N1—Mn1—N3—C6	67.3 (4)	Mn2—N5—C9—C8	172.1 (5)
Cl1—Mn1—N3—C6	162.5 (4)	C12—N7—C10—C11	−2.1 (9)
Cl3—Mn1—N3—C6	−23.4 (4)	Mn2—N7—C10—C11	178.9 (5)
Cl2^i^—Mn1—N3—C6	−107.6 (4)	N8—C11—C10—N7	1.7 (10)
N7—Mn2—N5—C7	72.7 (4)	C10—C11—N8—C12	−0.6 (10)
Cl4—Mn2—N5—C7	167.2 (4)	C11—N8—C12—N7	−0.6 (9)
Cl3—Mn2—N5—C7	−100.8 (4)	C10—N7—C12—N8	1.6 (9)
Cl2^i^—Mn2—N5—C7	−16.7 (4)	Mn2—N7—C12—N8	−179.3 (5)

Symmetry codes: (i) −*x*, −*y*, −*z*+1.

### Hydrogen-bond geometry (Å, °)

**Table:**

*D*—H···*A*	*D*—H	H···*A*	*D*···*A*	*D*—H···*A*
N8—H8···O1^ii^	0.88	2.09	2.865 (15)	147.
N4—H6A···Cl3^iii^	0.88	2.49	3.289 (4)	152.
N6—H10A···Cl4^iii^	0.88	2.48	3.247 (4)	146.
N2—H2A···Cl4^iv^	0.88	2.55	3.292 (4)	143.

Symmetry codes: (ii) *x*, *y*−1, *z*; (iii) *x*+1, *y*, *z*; (iv) −*x*, −*y*+1, −*z*+1.
